# Growth and characterization of a new inorganic metal–halide crystal structure, InPb_2_Cl_5_

**DOI:** 10.1107/S2056989023007892

**Published:** 2023-09-26

**Authors:** Michael P. Lewis, Ramjee Kandel, Gabriele Schatte, Peng L. Wang

**Affiliations:** a Department of Chemistry, Queen’s University, Kingston, Ontario K7L 3N6, Canada; University of Kentucky, USA

**Keywords:** crystal structure, inorganic, InPb_2_Cl_5_, solid-state

## Abstract

A new solid-state inorganic compound, indium dilead penta­chloride, InPb_2_Cl_5_, was synthesized by melting InCl and PbCl_2_ in a vacuum-sealed quartz ampoule. The ampoule was heated to 793 K and then slowly cooled to room temperature to induce crystallization of InPb_2_Cl_5_. InPb_2_Cl_5_ crystallizes in the monoclinic crystal system adopting a space group of type *P*2_1_/*c*, which is isostructural with other metal halides such as RbPb_2_Cl_5_, KPb_2_Cl_5_ and TlPb_2_Cl_5_. The bulk InPb_2_Cl_5_ exhibits a metallic black/grey colour, allowing it to be separated from white/yellow PbCl_2_ crystals. Due to the incongruent nature of the compound, the pure bulk InPb_2_Cl_5_ was not obtained. The black/grey InPb_2_Cl_5_ crystals were characterized by powder and single-crystal X-ray diffraction. InPbCl_3_ was also explored, however the growth was unsuccessful.

## Abstract 2

A new solid-state inorganic compound, InPb_2_Cl_5_, was synthesized by melting InCl and PbCl_2_ in a vacuum-sealed quartz ampoule. Bulk InPb_2_Cl_5_ was separated from PbCl2 and characterized by single-crystal X-ray diffraction.

## 1. Chemical context

Indium lead chloride, InPb_2_Cl_5_ is a metal halide that has been studied as a new material to be used in optoelectronic semiconducting applications. Other isostructural metal halides that have the structure *A*Pb_2_Cl_5_ (where *A* = K, Rb, Tl) have gained inter­est in fields such as optoelectronics (Vu *et al.*, 2020 ▸), and photovoltaics as a tunable laser (Isaenko *et al.*, 2013 ▸; Khyzhun *et al.*, 2014 ▸; Brown *et al.*, 2013 ▸). There has been success in growing metal-halide semiconducting crystals such as RbPb_2_Cl_5_ and KPb_2_Cl_5_ (Isaenko *et al.*, 2013 ▸; Rowe *et al.*, 2014 ▸; Isaenko *et al.*, 2009 ▸), however single-crystal InPb_2_Cl_5_ has not been reported and has only been computationally studied as the InPbCl_3_ phase (Khan *et al.*, 2022 ▸). Similar to RbPb_2_Cl_5_, KPb_2_Cl_5_, and TlPb_2_Cl_5_, InPb_2_Cl_5_ crystallizes in a monoclinic structure and has a space group of type *P*2_1_/*c*. Bulk InPb_2_Cl_5_ samples were prepared, which contained a mixture of black/grey metallic polycrystalline InPb_2_Cl_5_ at the bottom of the ampoule and white/yellow PbCl_2_ crystals above. When the black/grey crystals were broken up, the crystals appeared to have a much clearer and lighter green hue. The broken-up crystal was examined under an optical microscope and clear colourless crystal pieces were seen. The clear colourless single-crystal pieces were handpicked and characterized by single-crystal X-ray diffraction (XRD). The bulk material was ground using a mortar and pestle and the powder was characterized by powder-XRD. The powder-XRD pattern had low intensity peaks of In_7_Cl_9_ and PbCl_2_ impurities, but matched closely with the InPb_2_Cl_5_ phase. When InPb_2_Cl_5_ was left in ambient conditions over four months, the bulk absorbed moisture over time and left a light-grey film around the bulk with moisture build up on the side of the material.

## 2. Structural commentary

**Figure 1:**
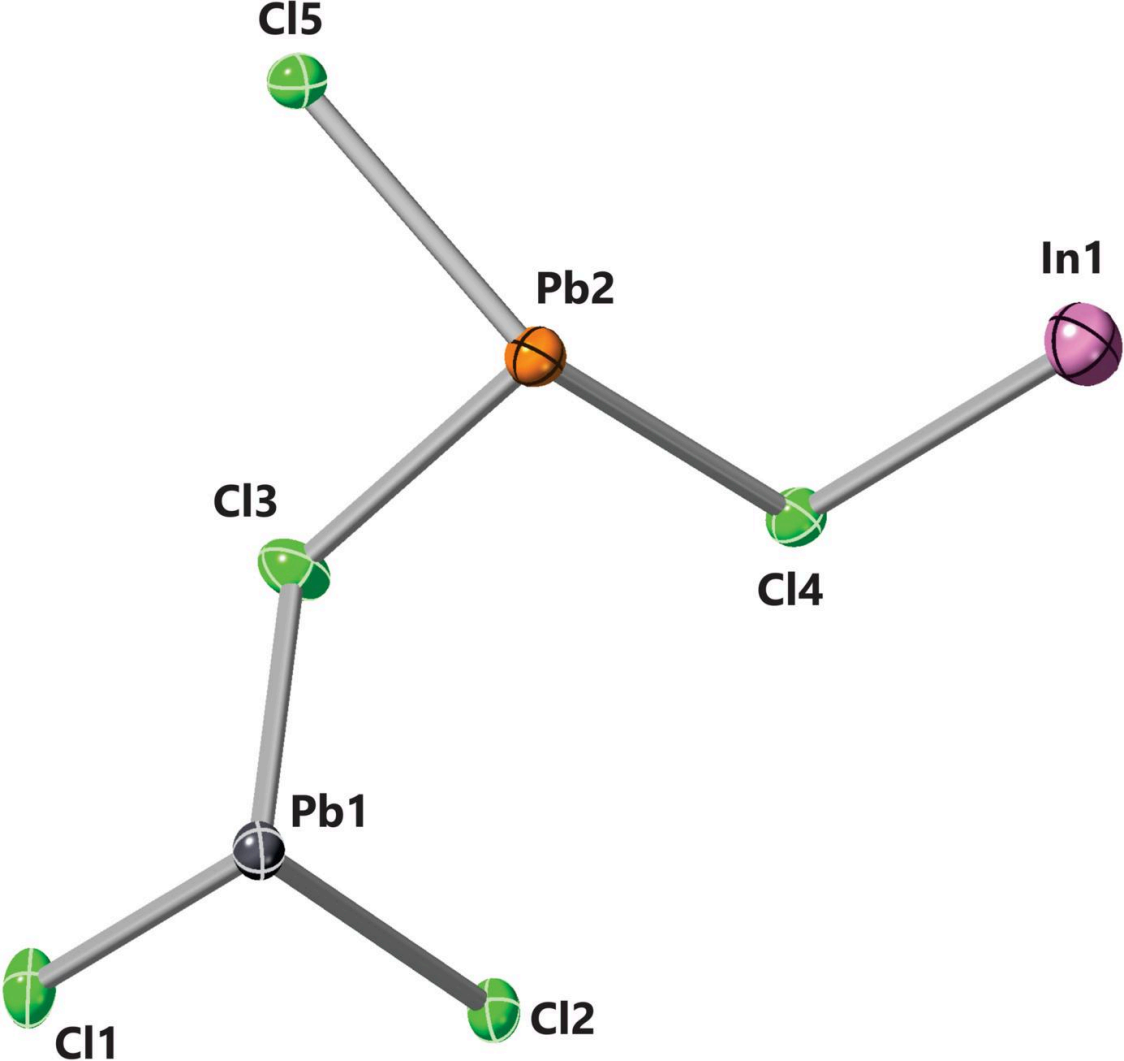
A view of the asymmetric unit. Ellipsoids are drawn at the 50% probability level.

**Figure 2:**
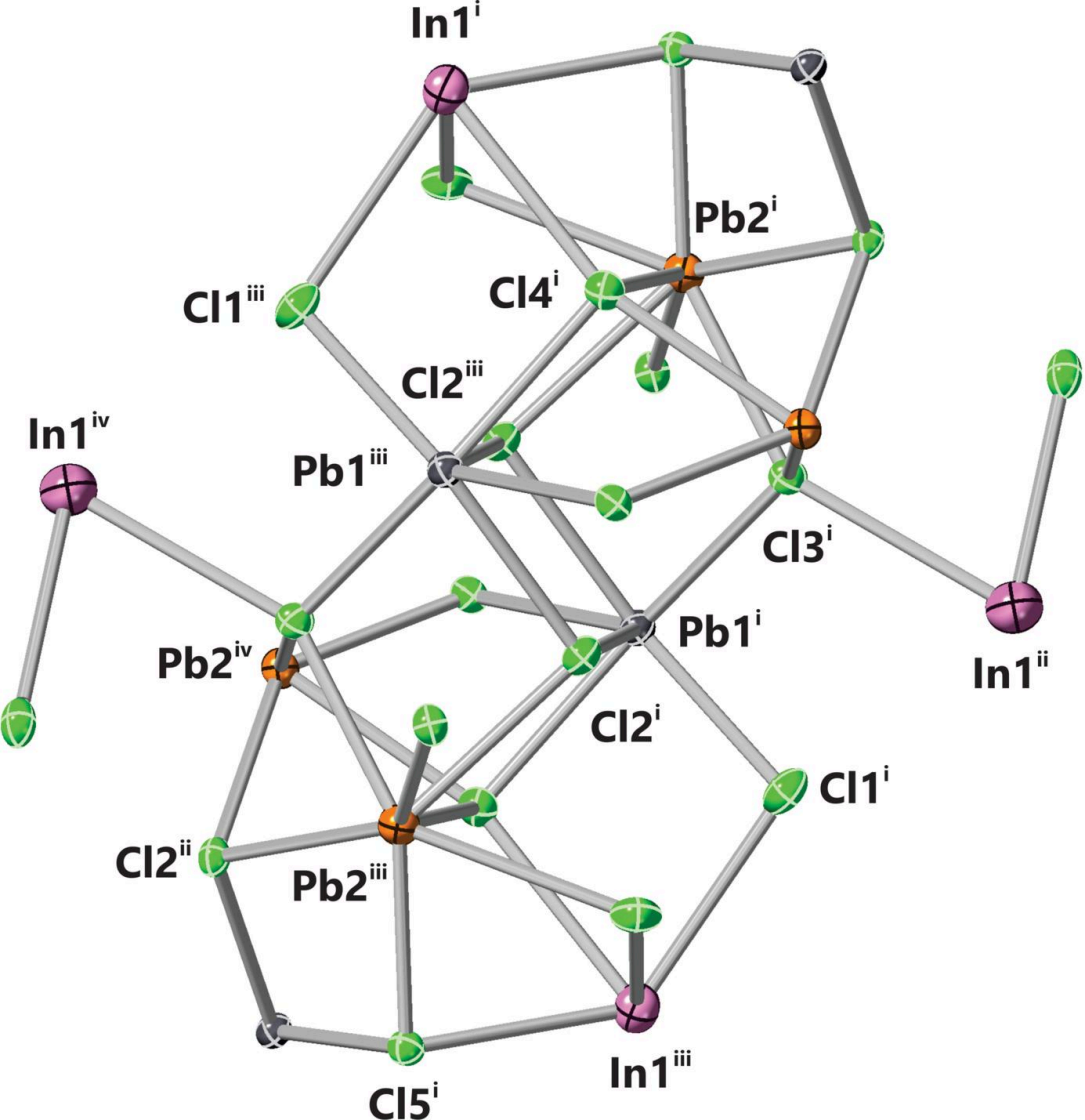
A partial packing plot viewed down the *b*-axis. Ellipsoids are drawn at the 50% probability level. Symmetry codes: (i) *x*, *y*, *z*; (ii) −*x*, *y* + 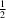 , −*z* + 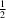 ; (iii) −*x*, −*y*, −*z*; (iv) *x*, −*y* − 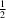 , *z* − 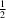 .

**Figure 3:**
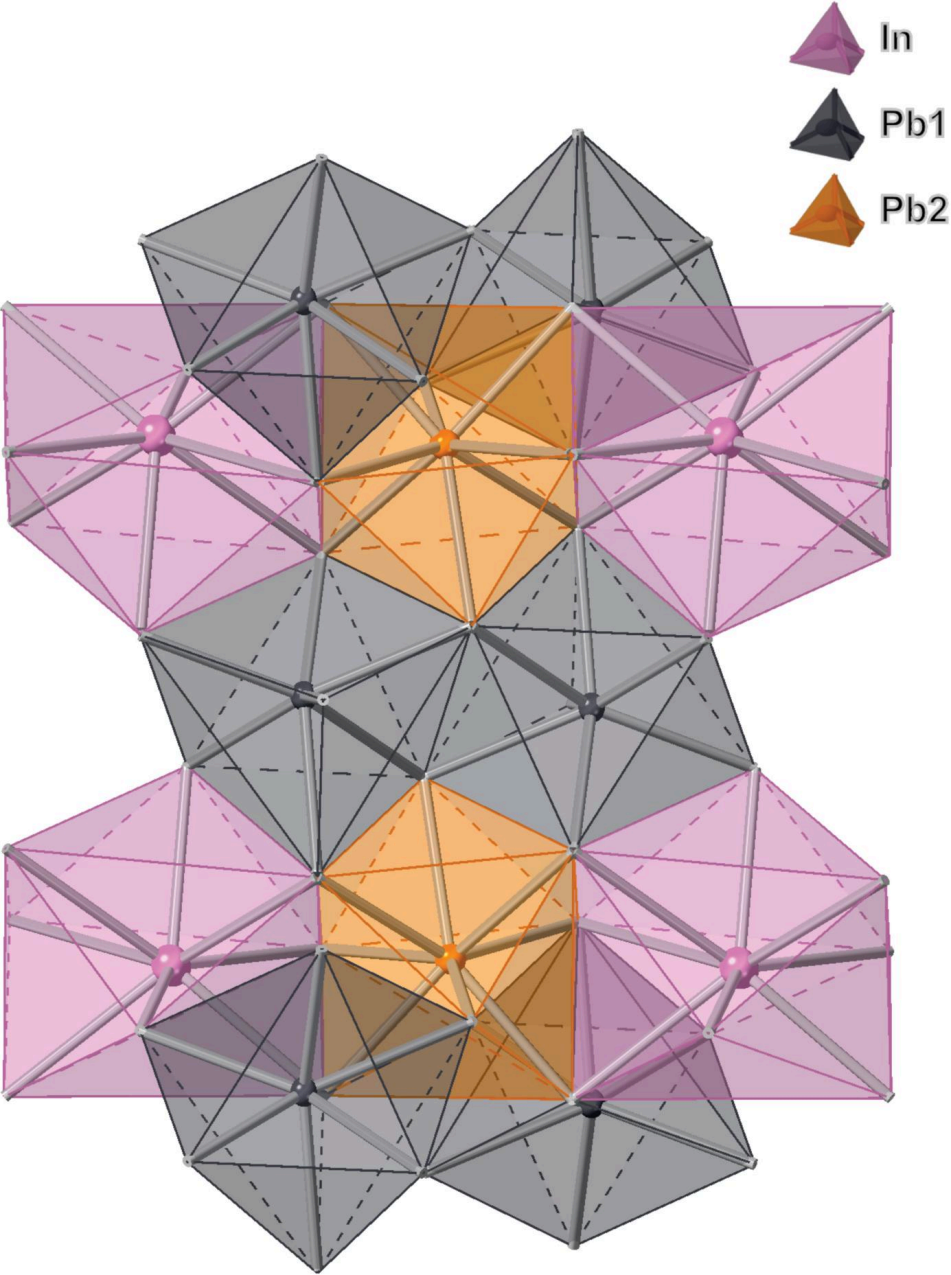
A polyhedron view of the crystal structure packing, viewed down the *b*-axis.

The single-crystal structure of InPb_2_Cl_5_ was found to adopt a monoclinic *P*2_1_/*c* space group. The single-crystal structure refinement confirmed the composition as InPb_2_Cl_5_. The bond lengths in the asymmetric unit (Fig. 1 ▸) of InPb_2_Cl_5_ are listed in Table 1 ▸. The unit cell of InPb_2_Cl_5_ (Fig. 2 ▸) has four symmetry-related formula units. The Pb atoms in the unit cell (Fig. 2 ▸) coordinate multiple chlorine atoms that give a range of bond lengths from 2.868 (5)–3.3145 (15) Å. The Pb1 atoms coordinate with seven chlorine atoms in the structure, with bond lengths ranging from 2.868 (5)–3.1371 (14) Å. The Pb1 atoms form a nine-face polyhedron with a volume of 37.374 Å^3^ (Fig. 3 ▸). The Pb2 atoms have a coordination number of 8 with bond lengths from 2.916 (7)–3.3145 (15) Å. The Pb2 atoms form a 12-face dodeca­hedron with a volume of 49.796 Å^3^ (Fig. 3 ▸). The shortest bond length is between Cl1 and Pb1, which is 2.868 (5) Å. The largest bond lengths are between the Pb2 atom and a Cl3 atom at 3.3145 (15) Å. The typical bond length between Pb and Cl atoms is 2.44 Å in the binary structure. There is an increase in bond lengths from the binary PbCl_2_ to the InPb_2_Cl_5_ structure. The indium atom inter­stitially coordinates eight chlorine atoms in a distorted octa­hedral geometry. The range of indium–chlorine bonds range from 3.1447 (18)–3.588 (8) Å. The indium atom forms a 12-face dodeca­hedron with a volume of 62.568 Å^3^ (Fig. 3 ▸). The typical In—Cl bond length is around 2.56 Å, indicating that the indium–chlorine bonds have a much weaker inter­action in the InPb_2_Cl_5_ structure. The largest bond angles seen in the unit cell (Fig. 2 ▸) are between the Cl1^iii^—Pb1—Cl2 atoms at 156.02 (3)° (symmetry codes as per Fig. 2 ▸). The shortest bond angle in the structure is between the Cl4^ii^—In1—Cl5^i^ atoms at 63.2587 (3)°. The Pb atoms have stronger inter­actions with the chlorine atoms resulting in shorter bond lengths and a wide range of bond lengths from 69.7087 (3)–156.02 (3)°. The indium atoms have weaker inter­actions and are inter­stitially located throughout the structure. The weaker inter­actions of the indium atoms is evident because of the shorter bond lengths and smaller range of bond angles from 63.2587 (3)–144.1653 (3)°. A complete list of bond lengths and bond angles is given in the supporting information.

## 3. Database survey

The InPb_2_Cl_5_ structure is isostructural with other compounds such as RbPb_2_Cl_5_ (Isaenko *et al.*, 2013 ▸; Isaenko *et al.*, 2009 ▸), KPb_2_Cl_5_ (Rowe *et al.*, 2014 ▸; Isaenko *et al.*, 2009 ▸) and TlPb_2_Cl_5_ (Khyzhun *et al.*, 2014 ▸). The cell dimensions of the monoclinic InPb_2_Cl_5_ cell are compared with the isostructural compounds in Table 2 ▸. The cell dimensions for InPb_2_Cl_5_ match very closely with TlPb_2_Cl_5_. There is no significant difference between the InPb_2_Cl_5_ structure and the isostructural structures in Table 2 ▸, the small difference is due to the atomic size difference for indium. The indium atom has the smallest atom size, so it is expected to fit tighter into the unit cell compared to the other structures, so we see that InPb_2_Cl_5_ has the smallest unit cell volume. The thallium atom is most comparable to the indium atom size, which is why its cell dimensions are most similar.

## 4. Synthesis and crystallization

Stoichiometric amounts of the binary compounds In^I^Cl (4g, ThermoFisher Scientific 99.995%, metals basis) and Pb^II^Cl_2_ [3.7278 (5)g, Acros Organics 99%] were mixed in a glove box under an argon environment (<0.1ppm O_2_ and H_2_O). The binary compounds were ground together with a mortar and pestle and then loaded into a quartz ampoule. The quartz ampoule was flame sealed under high vacuum (5.5 × 10^−5^ mbar). The loaded quartz ampoule was heated at 3K min^−1^ in a vertical furnace to 793K. The ampoule was cooled at 0.5 K min^−1^ to room temperature. A 1 mm^3^ piece of the metallic black and grey crystal was separated from the excess yellow Pb^II^Cl_2_ crystals and sent for characterization by powder-XRD and single-crystal XRD.

## 5. Refinement

The crystallographic data, data collection and structure refinement are summarized in Table 3 ▸.

## Supplementary Material

Crystal structure: contains datablock(s) global, I. DOI: 10.1107/S2056989023007892/pk2694sup1.cif

Structure factors: contains datablock(s) I. DOI: 10.1107/S2056989023007892/pk2694Isup2.hkl

CCDC reference: 2294068

Additional supporting information:  crystallographic information; 3D view; checkCIF report

## Figures and Tables

**Table 1:** Bond lengths (Å) in the InPb_2_Cl_5_ asymmetric unit (Fig. 1 ▸)

Bond	Distance
Cl1—Pb1	2.8677 (12)
Cl2—Pb1	2.9214 (10)
Cl3—Pb1	2.8744 (12)
Cl3—Pb2	2.9156 (12)
Cl4—Pb2	2.9236 (11)
Cl5—Pb2	2.9760 (12)

**Table 2:** InPb_2_Cl_5_ unit-cell parameters compared with isostructural compounds.

Compound	*a* (Å)	*b* (Å)	*c* (Å)	β (°)	Volume (Å^3^)
InPb_2_Cl_5_	8.9681 (11)	7.9033 (9)	12.4980 (16)	90.254 (6)	885.82 (19)
TlPb_2_Cl_5_	8.9561	7.9204	12.4908	90.073	886.0
RbPb_2_Cl_5_	8.9900	7.9963	12.541	90.20	901.5
KPb_2_Cl_5_	8.864	7.932	12.491	90.153	878.2

**Table 3:** Experimental details

Crystal data	
Chemical formula	In_2_Pb_4_Cl_10_
*M*_r_	1412.9
Crystal system, space group	Monoclinic, *P*2_1_/*c*
Temperature (K)	298
*a*, *b*, *c* (Å)	8.9681 (11), 7.9033 (9), 12.4980 (16)
β (°)	90.254 (6)
*V* (Å^3^)	885.82 (19)
*Z*	2
Radiation type	Mo *K*α
μ (mm^−1^)	41.91
Crystal size (mm)	0.22 × 0.18 × 0.13

Data collection	
Diffractometer	Bruker APEXII CCD
Absorption correction	Multi-scan (*SADABS*; Krause *et al.*, 2015 ▸)
*T*_min_, *T*_max_	0.378, 0.746
No. of measured, independent and observed [*I* > 2σ(*I*)] reflections	20682, 2699, 2423
*R*_int_	0.051
(sin θ/λ)_max_ (Å^−1^)	0.715

Refinement	
*R*[*F* ^2^ > 2σ(*F* ^2^)], *wR*(*F* ^2^), *S*	0.021, 0.042, 1.08
No. of reflections	2699
No. of parameters	74
Δρ_max_, Δρ_min_ (e Å^−3^)	1.22, −1.13

Computer programs: *APEX2* and *SAINT* (Bruker, 2012 ▸), *SHELXT* (Sheldrick, 2015*a* ▸), *SHELXL* (Sheldrick, 2015*b* ▸), *CrystalMaker* (Palmer, 2014 ▸), *WinGX* (Farrugia, 2012 ▸) and *publCIF* (Westrip, 2010 ▸).

## supplementary crystallographic information

### Crystal data

**Table:**

In_2_Pb_4_Cl_10_	*F*(000) = 1192
*M**_r_* = 1412.9	*D*_x_ = 5.297 Mg m^−^^3^
Monoclinic, *P*2_1_/*c*	Mo *K*α radiation, λ = 0.71073 Å
Hall symbol: -P 2ybc	Cell parameters from 9997 reflections
*a* = 8.9681 (11) Å	θ = 2.3–30.5°
*b* = 7.9033 (9) Å	µ = 41.91 mm^−^^1^
*c* = 12.4980 (16) Å	*T* = 298 K
β = 90.254 (6)°	Transparent square, colourless
*V* = 885.82 (19) Å^3^	0.22 × 0.18 × 0.13 mm
*Z* = 2	

### Data collection

**Table:**

Bruker APEXII CCD diffractometer	2699 independent reflections
Radiation source: sealed X-ray tube, Incoatec Iµs	2423 reflections with *I* > 2σ(*I*)
Graphite monochromator	*R*_int_ = 0.051
Detector resolution: 7.9 pixels mm^-1^	θ_max_ = 30.6°, θ_min_ = 2.3°
φ and ω scans	*h* = −12→12
Absorption correction: multi-scan (SADABS; Krause *et al.*, 2015)	*k* = −11→11
*T*_min_ = 0.378, *T*_max_ = 0.746	*l* = −17→17
20682 measured reflections	

### Refinement

**Table:**

Refinement on *F*^2^	0 constraints
Least-squares matrix: full	Primary atom site location: structure-invariant direct methods
*R*[*F*^2^ > 2σ(*F*^2^)] = 0.021	*w* = 1/[σ^2^(*F*_o_^2^) + (0.0083*P*)^2^ + 1.8133*P*] where *P* = (*F*_o_^2^ + 2*F*_c_^2^)/3
*wR*(*F*^2^) = 0.042	(Δ/σ)_max_ = 0.002
*S* = 1.08	Δρ_max_ = 1.22 e Å^−^^3^
2699 reflections	Δρ_min_ = −1.13 e Å^−^^3^
74 parameters	Extinction correction: SHELXL-2018/3 (Sheldrick 2015b), Fc^*^=kFc[1+0.001xFc^2^λ^3^/sin(2θ)]^-1/4^
0 restraints	Extinction coefficient: 0.00232 (8)

### Special details

**Table:**

Geometry. All esds (except the esd in the dihedral angle between two l.s. planes) are estimated using the full covariance matrix. The cell esds are taken into account individually in the estimation of esds in distances, angles and torsion angles; correlations between esds in cell parameters are only used when they are defined by crystal symmetry. An approximate (isotropic) treatment of cell esds is used for estimating esds involving l.s. planes.

### Fractional atomic coordinates and isotropic or equivalent isotropic displacement parameters (Å^2^)

**Table:**

	*x*	*y*	*z*	*U*_iso_*/*U*_eq_	
Cl1	0.04109 (12)	0.67574 (15)	0.41739 (11)	0.0316 (3)	
Cl2	0.45758 (11)	0.66555 (13)	0.40434 (9)	0.0226 (2)	
Cl3	0.27835 (14)	0.65746 (14)	0.68735 (10)	0.0304 (3)	
Cl4	0.72918 (12)	0.68702 (14)	0.72264 (9)	0.0252 (2)	
Cl5	0.28105 (12)	0.45943 (14)	1.00167 (9)	0.0232 (2)	
In1	0.98678 (5)	0.45346 (6)	0.83468 (4)	0.04410 (11)	
Pb1	0.24664 (2)	0.43551 (2)	0.50647 (2)	0.02229 (6)	
Pb2	0.49498 (2)	0.48805 (2)	0.82509 (2)	0.02538 (6)	

### Atomic displacement parameters (Å^2^)

**Table:**

	*U*^11^	*U*^22^	*U*^33^	*U*^12^	*U*^13^	*U*^23^
Cl1	0.0194 (5)	0.0309 (6)	0.0445 (7)	0.0009 (4)	0.0017 (5)	0.0137 (5)
Cl2	0.0192 (5)	0.0201 (4)	0.0285 (6)	−0.0008 (4)	−0.0002 (4)	0.0059 (4)
Cl3	0.0364 (6)	0.0283 (5)	0.0263 (6)	0.0084 (5)	−0.0081 (5)	−0.0063 (4)
Cl4	0.0259 (5)	0.0254 (5)	0.0243 (6)	−0.0027 (4)	0.0021 (4)	0.0025 (4)
Cl5	0.0251 (5)	0.0240 (5)	0.0205 (5)	0.0004 (4)	0.0005 (4)	0.0009 (4)
In1	0.0418 (2)	0.0432 (2)	0.0472 (3)	0.00874 (18)	−0.00359 (19)	−0.00495 (19)
Pb1	0.01876 (9)	0.02412 (9)	0.02399 (10)	−0.00042 (6)	−0.00025 (6)	−0.00142 (6)
Pb2	0.02614 (10)	0.02526 (9)	0.02473 (10)	−0.00304 (7)	0.00037 (7)	0.00179 (6)

### Geometric parameters (Å, º)

**Table:**

Cl1—Pb1	2.8677 (12)	Cl3—Pb2	2.9156 (12)
Cl1—Pb1^i^	2.8912 (11)	Cl4—Pb2	2.9236 (11)
Cl2—Pb1	2.9214 (10)	Cl4—Pb1^iii^	3.0314 (12)
Cl2—Pb2^ii^	2.9311 (10)	Cl5—Pb2	2.9394 (11)
Cl2—Pb1^iii^	2.9817 (11)	Cl5—Pb2^iv^	2.9760 (12)
Cl3—Pb1	2.8744 (12)		
			
Pb1—Cl1—Pb1^i^	104.12 (4)	Cl2—Pb1—Cl2^iii^	75.72 (3)
Pb1—Cl2—Pb2^ii^	143.53 (4)	Cl1—Pb1—Cl4^iii^	83.88 (4)
Pb1—Cl2—Pb1^iii^	104.28 (3)	Cl3—Pb1—Cl4^iii^	158.52 (3)
Pb2^ii^—Cl2—Pb1^iii^	105.88 (3)	Cl1^i^—Pb1—Cl4^iii^	106.34 (4)
Pb1—Cl3—Pb2	104.33 (4)	Cl2—Pb1—Cl4^iii^	74.75 (3)
Pb2—Cl4—Pb1^iii^	107.24 (4)	Cl2^iii^—Pb1—Cl4^iii^	101.55 (3)
Pb2—Cl5—Pb2^iv^	95.44 (3)	Cl3—Pb2—Cl4	88.42 (4)
Cl1—Pb1—Cl3	87.85 (4)	Cl3—Pb2—Cl2^v^	72.16 (3)
Cl1—Pb1—Cl1^i^	75.88 (4)	Cl4—Pb2—Cl2^v^	74.28 (3)
Cl3—Pb1—Cl1^i^	90.69 (4)	Cl3—Pb2—Cl5	92.48 (3)
Cl1—Pb1—Cl2	80.49 (3)	Cl4—Pb2—Cl5	147.44 (3)
Cl3—Pb1—Cl2	84.37 (4)	Cl2^v^—Pb2—Cl5	75.06 (3)
Cl1^i^—Pb1—Cl2	156.02 (3)	Cl3—Pb2—Cl5^iv^	144.23 (3)
Cl1—Pb1—Cl2^iii^	153.09 (3)	Cl4—Pb2—Cl5^iv^	76.10 (3)
Cl3—Pb1—Cl2^iii^	77.57 (3)	Cl2^v^—Pb2—Cl5^iv^	72.65 (3)
Cl1^i^—Pb1—Cl2^iii^	126.11 (3)	Cl5—Pb2—Cl5^iv^	84.56 (3)

Symmetry codes: (i) −*x*, −*y*+1, −*z*+1; (ii) *x*, −*y*+3/2, *z*−1/2; (iii) −*x*+1, −*y*+1, −*z*+1; (iv) −*x*+1, −*y*+1, −*z*+2; (v) *x*, −*y*+3/2, *z*+1/2.
